# Drugs for treating myocardial fibrosis

**DOI:** 10.3389/fphar.2023.1221881

**Published:** 2023-09-12

**Authors:** Zhanhong Ren, Zixuan Zhang, Li Ling, Xiufen Liu, Xin Wang

**Affiliations:** ^1^ Hubei Key Laboratory of Diabetes and Angiopathy, Medicine Research Institute, Xianning Medical College, Hubei University of Science and Technology, Xianning, China; ^2^ School of Basic Medical Sciences, Xianning Medical College, Hubei University of Science and Technology, Xianning, China; ^3^ School of Mathematics and Statistics, Hubei University of Science and Technology, Xianning, China

**Keywords:** myocardial fibrosis, modern medicine, traditional Chinese medicine, integrated traditional Chinese and modern medicine, mechanism

## Abstract

Myocardial fibrosis, which is a common pathological manifestation of many cardiovascular diseases, is characterized by excessive proliferation, collagen deposition and abnormal distribution of extracellular matrix fibroblasts. In clinical practice, modern medicines, such as diuretic and β receptor blockers, and traditional Chinese medicines, such as *salvia miltiorrhiza* and safflower extract, have certain therapeutic effects on myocardial fibrosis. We reviewed some representative modern medicines and traditional Chinese medicines (TCMs) and their related molecular mechanisms for the treatment of myocardial fibrosis. These drugs alleviate myocardial fibrosis by affecting related signaling pathways and inhibiting myocardial fibrosis-related protein synthesis. This review will provide more references and help for the research and treatment of myocardial fibrosis.

## 1 Introduction

**FIGURE 1:**
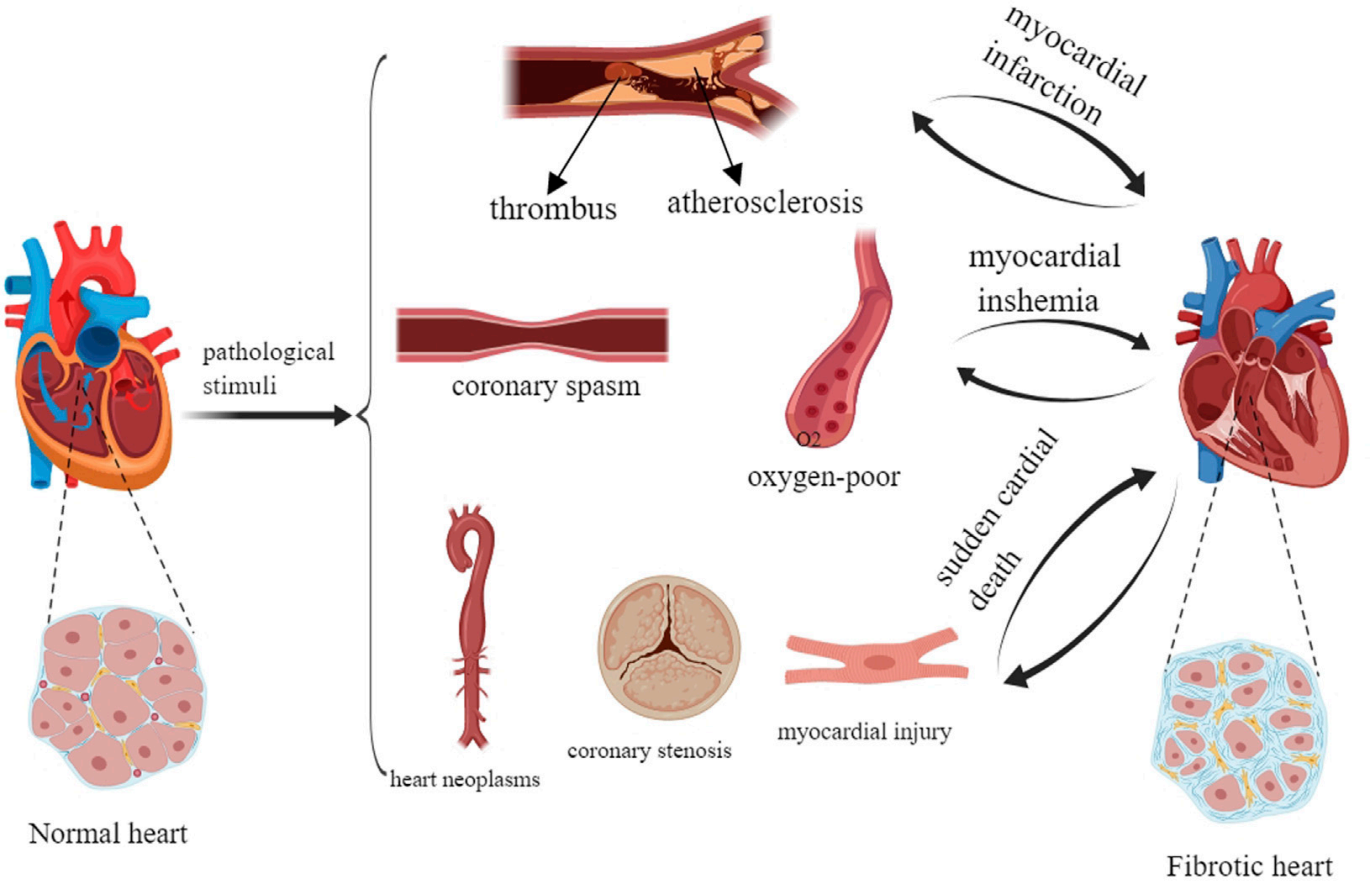
Myocardial fibrosis.

Myocardial fibrosis (MF) is a common pathological change in various heart diseases such as atherosclerosis and coronary disease, and the main pathological feature of myocardial infarction. An injury in the vessel wall during atherosclerosis can promote transforming growth factor-β (TGF-β) production by vascular and inflammatory cells, which can mediate fibrotic and inflammatory components in the lesion and lead to MF (Goumans and Ten Dijke, 2018). Coronary disease leads to MF by increasing the levels of type I and type III collagen (Ismail et al., 1999). In myocardial infarction, the persistent activated myofibroblasts in the infarct scar can continuously produce profibrotic factors that translocate to remote areas of the myocardium, which can result in the activation and proliferation of local fibroblasts and promote interstitial and perivascular fibrosis (Talman and Ruskoaho, 2016). In addition, mechanical stress in the undamaged left ventricular wall may be a risk factor for MF (Talman and Ruskoaho, 2016). According to an epidemiological survey, more than 800,000 people worldwide die of fibrous diseases, especially pulmonary and cardiac fibrosis, every year. MF is the response mechanism of myocardial injury. Its main pathological features are the proliferation and activation of cardiac fibroblasts (CFs) (Cheng et al., 2023), the deposition of extracellular matrix (ECM), the formation of scar tissue, a decrease in tissue compliance and a decline in cardiac function (Ren et al., 2022). MF is not only the pathological reaction of cardiovascular diseases including myocardial infarction, myocardial ischemia and sudden cardiac death, but also the risk factor for their further deterioration (Talman and Ruskoaho, 2016; Ambrose, 2006; Torrisi et al., 2020) (Figure 1). In recent years, studies on modern and traditional Chinese medicine (TCM) for the treatment of MF have been carried out, and a series of achievements in drug treatment have been obtained (González et al., 2018) (Table 1).

**TABLE 1:** Categories of drugs for treating MF.

	classification	name	Reference
Modern medicine	ACEI	Perindopril, Captopril	Schalekamp et al. (1980), Yoshimura et al. (1989), Plosker and McTavish (1995), Howes and Christie (1998), Dendorfer et al. (2005), Jessup et al. (2009), Li et al. (2019), Zhang et al. (2019), Blanda et al. (2020), Liu et al. (2021), Rha et al. (2021), Slack et al. (2021), Akhtar et al. (2022), Hu et al. (2022), Li et al. (2022)
	Diuretic	Eplerenone, Torsemide, Canrenoate Potassium	Bos et al. (2004), López et al. (2004), Bos et al. (2005), López et al. (2007), Struthers et al. (2008), Adam et al. (2015), Chen et al. (2016), Kurlykina et al. (2017), Shao et al. (2018), Diuretics (2021), Greene et al. (2021), Rossier (2021), Buffolo et al. (2022), Schimmel et al. (2022)
	β -receptor blocker	Propranolol, Carvedilol	Pacca et al. (2002), Czuriga and Edes (2004), Perlini et al. (2005), Zhu et al. (2013), Diuretics (2018), Nuamnaichati et al. (2018), Oliver et al. (2019), Zheng et al. (2019), Ibrahim et al. (2020), Ibrahim et al. (2021), Tsai et al. (2022), Ke et al. (2023)
Traditional Chinese medicine	Salvia miltiorrhiza and Carthamus tinctorius	Danshinone, Safflower yellow, Salvia miltiorrhiza safflower	Jiang et al. (2019), Yang et al. (2019), Bai et al. (2020), Orgah et al. (2020), Wang et al. (2020), Yang et al. (2023)
	Astragaloside	Astragaloside IV, Astragalus saponins	Lu et al. (2017), Wan et al. (2018), Tan et al. (2020), Wei et al. (2020), Zhang et al. (2022), Zhu et al. (2022), Ren et al. (2023)
	Angesica	Angelica sinensis polysacchardie, butylphthalide	Pan and Zhu (2018), Lin et al. (2019), Chang et al. (2021), Song et al. (2021)

There is a long history of using modern medicine to treat MF. The development of anti-myocardial fibrosis drugs has decreased the case fatality rate for MF (Ghionzoli et al., 2022). The occurrence and development of MF are closely linked with the renin-angiotensin-aldosterone system (RAAS), oxidative stress, immune inflammation, the matrix metalloproteinase system, fibroblast proliferation and the TGF-β1/Smad3 signaling pathway [^5^]. Some drugs that target these pathways, such as RAAS inhibitors, have been proven to be effective in reducing ECM deposition in the myocardium (Friedman, 2022). However, MF is characterized by rapid onset, high mortality and complicated mechanisms, and effective treatments for MF via modern medicine are lacking. To date, no primarily antifibrotic drugs have been approved for the treatment of cardiovascular disease (Morfino et al., 2023). Although many prospective targets in the treatment of MF have been discovered, there is still no evidence of clinical benefits [^11^].

TCM has a long history of active ingredients, extracts, and herbal formulas that are produced by boiling, frying and other processing methods to treat human diseases (Li et al., 2021). Emerging evidence has demonstrated that bioactive ingredients in TCM have multiple antifibrotic effects; thus, TCM is recognized as an important and effective treatment strategy for MF (Li et al., 2021). TCM has good therapeutic potential for treating MF with low costs and side effects (Li et al., 2023). In addition, due to its multicomponent, multitarget and multilevel characteristics, TCM can also be used to treat different fibrotic and cardiovascular diseases in different stages (Li et al., 2023). However, it has some shortcomings. The specific effective ingredients in TCM and related molecular mechanisms are not clear, and there is a relatively weak theoretical and scientific basis for the use of TCM. Although there are various TCM methods for the treatment of MF, comparative pharmacological studies are lacking (Zhang et al., 2023). Further study is needed to elucidate the molecular mechanisms underlying the prevention and treatment of MF by TCM (Zhang et al., 2023). Clinically, a unified TCM syndrome differentiation system is also lacking because symptoms and manifestations vary with each individual (Ren et al., 2022). In addition, the production of TCM needs stricter and more standard regulation and quality control to improve the quality, purity and potency of TCM drugs.

Here, we reviewed some representative drugs and their molecular mechanisms. This article will provide more references and lay a foundation for further research on the treatment of MF.

A normal heart can progressively develop and transform into a fibrotic heart. MF occurs during the pathological process of myocardial infarction, myocardial ischemia and sudden cardiac death. Severe MF can accelerate and worsen myocardial infarction, myocardial ischemia, and sudden cardiac death. Myocardial infarction is mainly caused by heart tumors, coronary artery stenosis and myocardial injury. Myocardial ischemia is mainly caused by coronary artery spasm and hypoxia. Sudden cardiac death is mainly caused by atherosclerosis.

Modern medicine is divided into three categories: angiotensin converting enzyme inhibitors (ACEIs), diuretics, and β-receiver blockers. The typical representative ACEIs include perindopril and captopril; typical representative diuretics include eplerenone, tolasemide, and canrenoate potassium; and typical representative β-receiver blockers include propranolol and carvedilol. TCM is divided into three categories: *Salvia miltiorrhiza* and *Carthamus tinctorius* extract, *astragaloside*, and *angesica*. The typical *Salvia miltiorrhiza* and *Carthamus tinctorius* extracts include danshinone, safflower yellow and *Salvia miltiorrhiza* safflower. The typical representative *astragalosides* include astragaloside Ⅳ and astragalus saponins. The typical representative *Angesica* drugs include angelica sinensis and polysaccharide.

## 2 Modern medicine

### 2.1 Angiotensin converting enzyme inhibitors (ACEIs)

**FIGURE 2:**
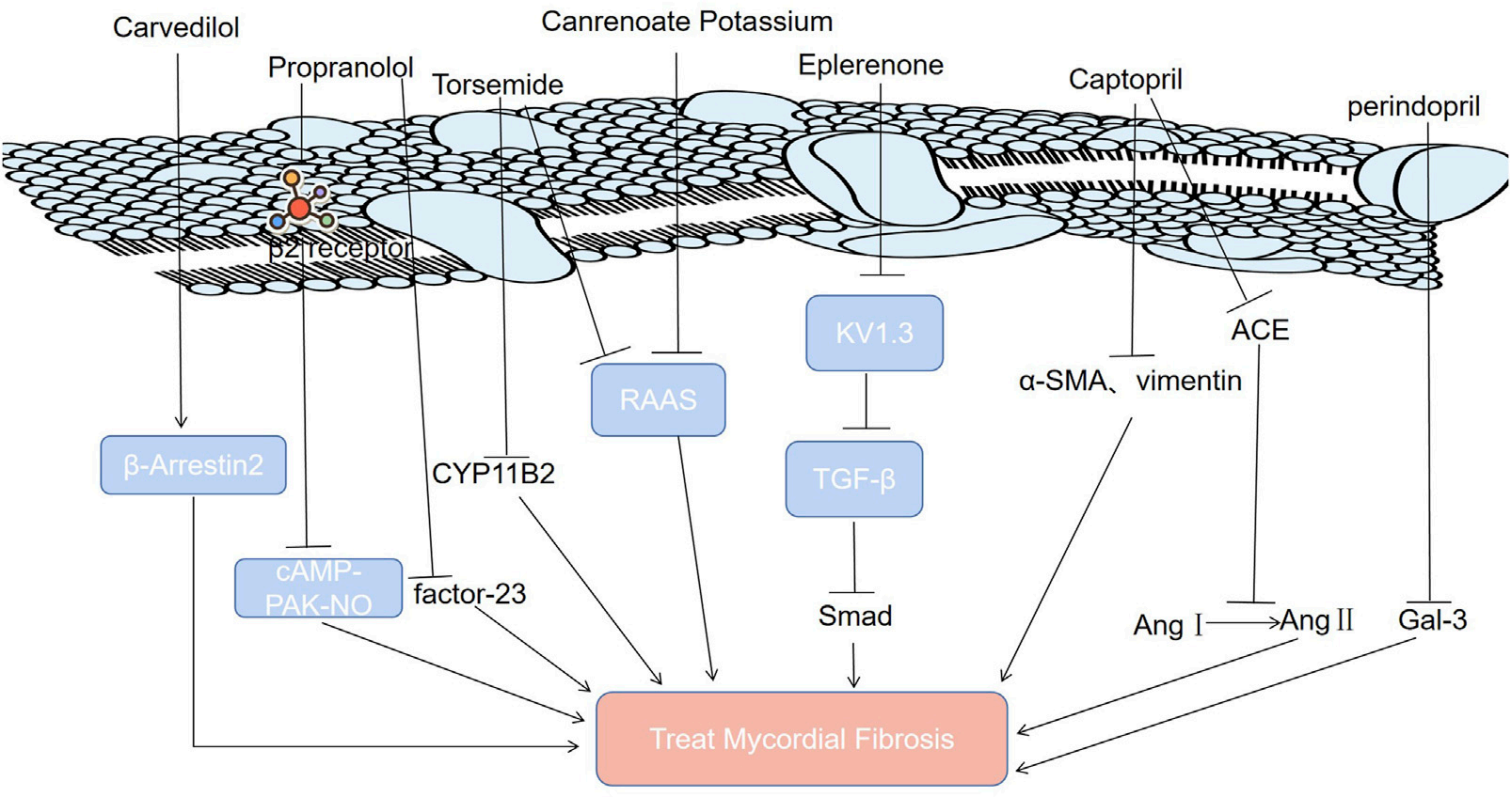
Molecular mechanisms of modern medicine for treating MF.

ACEIs are one of the most studied and effective drug types in the treatment of MF (Jessup et al., 2009). Blocking the renin angiotensin system (RAS) with ACEIs can prevent fibrosis development (Table 1) (Liu et al., 2021). Perindopril is a powerful and long-lasting ACEI (Li et al., 2019). Recent studies have shown that perindopril can alleviate MF by reducing the levels of galectin-3 (Gal-3) (Figure 2) (Li et al., 2019; Hu et al., 2022; Blanda et al., 2020; Slack et al., 2021). Captopril is a classic drug for the clinical treatment of MF (Yoshimura et al., 1989). It inhibits MF in two ways (Zhang et al., 2019; Plosker and McTavish, 1995; Schalekamp et al., 1980; Akhtar et al., 2022; Rha et al., 2021; Li et al., 2022). First, it can hinder the conversion of angiotensin I to angiotensin II by inhibiting ACE (Zhang et al., 2019; Plosker and McTavish, 1995; Schalekamp et al., 1980). It can improve ventricular remodeling and inhibit the occurrence of MF (Figure 2) (Akhtar et al., 2022). Second, captopril can inhibit MF by downregulating α-smooth muscle actin and vimentin expression (Rha et al., 2021; Li et al., 2022).

MF can result from cardiovascular diseases such as myocardial infarction and atherosclerosis. Although ACEI is the most commonly used antihypertensive drug, it is underutilized in the treatment of heart failure and left ventricular dysfunction after myocardial infarction and its application in treating atherosclerosis is limited (Howes and Christie, 1998; Dendorfer et al., 2005). In addition, the molecular mechanisms through which perindopril can alleviate interstitial collagen deposition in the myocardium and inhibit MF have not been elucidated, which hinders its application in the treatment of MF (Li et al., 2019).

### 2.2 Diuretics

It has been proven that the phenomenon of “aldosterone escape” determines the irreplaceable role of aldosterone antagonists in the treatment of MF (Table 1) (Buffolo et al., 2022; Schimmel et al., 2022; Rossier, 2021). It has been shown that eplerenone’s high affinity for Kv1.3 channels, potassium channel proteins on the T-lymphocyte (Treg) membrane, enables it to antagonize Kv1.3 channels directly to suppress the proliferation of Tregs that can secrete TGF-β (Shao et al., 2018; Chen et al., 2016). Therefore, eplerenone can alleviate MF (Shao et al., 2018; Chen et al., 2016). Canrenoate potassium, a new aldosterone receptor antagonist, has fewer side effects related to sex hormones than spironolactone (Bos et al., 2005). It alleviates isoproterenol-induced MF in rats by inhibiting the RAAS (Bos et al., 2004). The long-acting loop diuretic torsemide can antagonize aldosterone, antagonize the physiological effect of aldosterone on water sodium retention, inhibit the activation of the RAAS (Figure 2), and expel excess water from between tissues, which avoids the side effects of hyperactivity of the RAAS system caused by long-term use of diuretics (Kurlykina et al., 2017; Ghionzoli et al., 2022). Therefore, torsemide has a good cardioprotective effect that delays or reverses MF. In addition, inhibiting aldosterone synthase (CYP11B2) with torsemide prevents atrial fibrosis and atrial fibrillation in mice (Adam et al., 2015).

Diuretics, a type of the most frequently used medications, are well tolerated (Diuretics, 2021). Their common side effects resulting from the diuresis and mineral loss include weakness, dizziness, electrolyte imbalance, low sodium and low potassium (Diuretics, 2021). Aldosterone receptor antagonists have therapeutic effects on MF without increasing aldosterone or inducing sodium retention, but under other physiological conditions, it is unknown whether they also have the same potency (Rossier, 2021). Dose-dependent blood potassium elevation is an adverse reaction of eplerenone (Struthers et al., 2008). In addition, eplerenone is also associated with sexual side effects (Struthers et al., 2008). Torsemide has significant advantages in terms of diuretic effects and high bioavailability (Greene et al., 2021). Previous studies have proven that torsemide can inhibit MF and reverse ventricular remodeling (López et al., 2004; López et al., 2007). However, its detailed mechanisms remain unclear.

### 2.3 β receptor blockers

β receptor blockers can treat MF and other cardiovascular diseases (Ke et al., 2023). Hyperactivation of the sympathetic nerve is one of the common pathophysiological mechanisms of MF and many cardiovascular diseases (Table 1) (Czuriga and Edes, 2004; Perlini et al., 2005). β receptor blockers can inhibit the effects of epinephrine and norepinephrine on multiple tissues and systems, thereby inhibiting MF (Oliver et al., 2019).

Propranolol is a type of β receptor blocker (Tsai et al., 2022). It can cut off the β2 receptor and inhibit the cyclic adenosine phosphate (CAMP)/protein kinase A (PAK) nitric oxide signaling pathway to alleviate MF (Nuamnaichati et al., 2018). Propranolol inhibits MF by inhibiting the expression of fibroblast growth factor-23 (FGF-23) (Figure 2) (Tsai et al., 2022).

Carvedilol is the most effective way to improve the survival rate after myocardial infarction (Zheng et al., 2019). Carvedilol, a blocker of β1 and β2 adrenergic receptors (ARs), ameliorates MF induced by a high-fructose/high-fat diet in mice by enhancing cardiac β-arrestin 2 signaling (Figure 2) (Ibrahim et al., 2020; Ibrahim et al., 2021).

β receptor blockers are one of the most commonly used drugs and are usually well tolerated (Diuretics, 2018). However, β-adrenergic blockade can lead to common side effects, including bradycardia, fatigue, dizziness, depression, memory loss, insomnia, impotence and chills in the limbs (Diuretics, 2018). The mechanism by which propranolol can compensate for local nitric oxide deficiency in arterial circulation is still unclear, which limits its application (Pacca et al., 2002). Although carvedilol can inhibit MF, its exact mechanism remains unknown (Zhu et al., 2013).

### 2.4 Other modern medicines

Other modern medicines, such as irisin and clopidogrel, have therapeutic effects on MF (Pan et al., 2021; Jia et al., 2013). Irisin inhibits MF by inhibiting the levels of reactive oxygen species (ROS) and the NF-KB-Snail Signaling Pathway (Pan et al., 2021). Clopidogrel inhibits platelet activation, inhibits platelet leukocyte binding, and causes inflammatory cells to secrete cytokines (IL-1) into the heart, thus inhibiting MF (Table 2) (Jia et al., 2013; Liu et al., 2020).

**TABLE 2:** Molecular mechanisms by which other modern medicines and traditional Chinese medicines treat MF.

name	Mechanism	Reference
Irisin	Irisin―|ROS→NF-KB-Snail→Mycordial Fibrosis	Pan et al. (2021)
Clopidogrel	Clopidogrel―|platelet (activation)→Mycordial Fibrosis	(Jia et al., 2013; Liu et al., 2020)
Curcumin	Curcumin→SIRT1,Nrf2,NADPFoxidase subunits―|Mycordial Fibrosis	(Ren et al., 2022; Sadoughi et al., 2021)
Earthworm	earthworm―|ERK1/2,uPA,SP1, CTGF proteins→Mycordial Fibrosis	(Huang et al., 2019)]
Ginsenoside Re	ginsenoside―|miR-489/myd88/NF-KB→Mycordial Fibrosis	Sun et al. (2023)

### 2.5 Combined treatment of MF

Compared with single drug treatment of MF, the combined use of different types of drugs to treat MF is superior (Cohn, 2003). After myocardial infarction in rats, the therapeutic effect of the combination of eplerenone and the angiotensin Ⅱ receptor antagonist (ARB) candesartan was better than that of single drug therapy (Fraccarollo et al., 2003). The combination of β receptor blockers and ACEIs can provide a comprehensive neuroendocrine blocking effect, especially in the heart and blood vessels (Demkes et al., 2021).

Modern medicine inhibits MF by downregulating certain signaling pathways (TGF-β/Smad, cAMP-PAK-NO, RAAS), protein levels (factor-23, Gal-3, ACE) and receptors (β2 receptor). Gal-3: galectin-3; RAAS: renin-angiotensin-aldosterone system; Ang Ⅰ: angiotensin Ⅰ; Ang Ⅱ: angiotensin Ⅱ; TGF-β: transforming growth factor-β; β-Arrestin 2: a key protein regulating endothelial nitric oxide synthase activity; CYP11B2: aldosterone synthase; KV1.3: the Kv1.3 channel, a potassium channel protein on the membrane of T lymphocytes; cAMP-PAK-NO: cyclic adenosine phosphate (CAMP)/protein kinase A (PAK) nitric oxide signaling pathway; FGF-23: fibroblast growth factor-23; Smad: a TGF-β intracellular signaling molecules in the cytokine superfamily; α-SMA: α-smooth muscle actin; vimentin: a fibroblast marker; ACE: angiotensin converting enzyme.

## 3 Traditional Chinese medicine

### 3.1 *Salvia miltiorrhiza* and *Carthamus tinctorius* extract

*Salvia miltiorrhiza* and *Carthamus tinctorius* extracts (SCE) have been widely used in clinical practice and have achieved good effects in the treatment of myocardial ischemia and MF (Table 1) (Wang et al., 2020). The chalcone pigment safflower yellow (SY) is the main effective component of *Carthamus tinctorius* (Bai et al., 2020). SY can be used to treat atherosclerosis by reducing blood lipid levels and improving antioxidant capacity (Bai et al., 2020). Danshinone, an active component of *Salvia miltiorrhiza*, can ameliorate MF (Jiang et al., 2019). It has two mechanisms. First, danshinone has been suggested to reverse the increase in the levels of collagen type 1 (Col1), collagen type 3 (Col3) and α-smooth muscle actin (α-SMA) in HF rats induced by ligation of the left anterior descending branch (LAD) of the coronary artery through upregulating miR-205-3p, miR-29b, or miR-618 (Yang et al., 2023). Second, MF is achieved by downregulating the TGF-β/SMAD2/3 signaling pathway (Ren et al., 2022).

**FIGURE 3:**
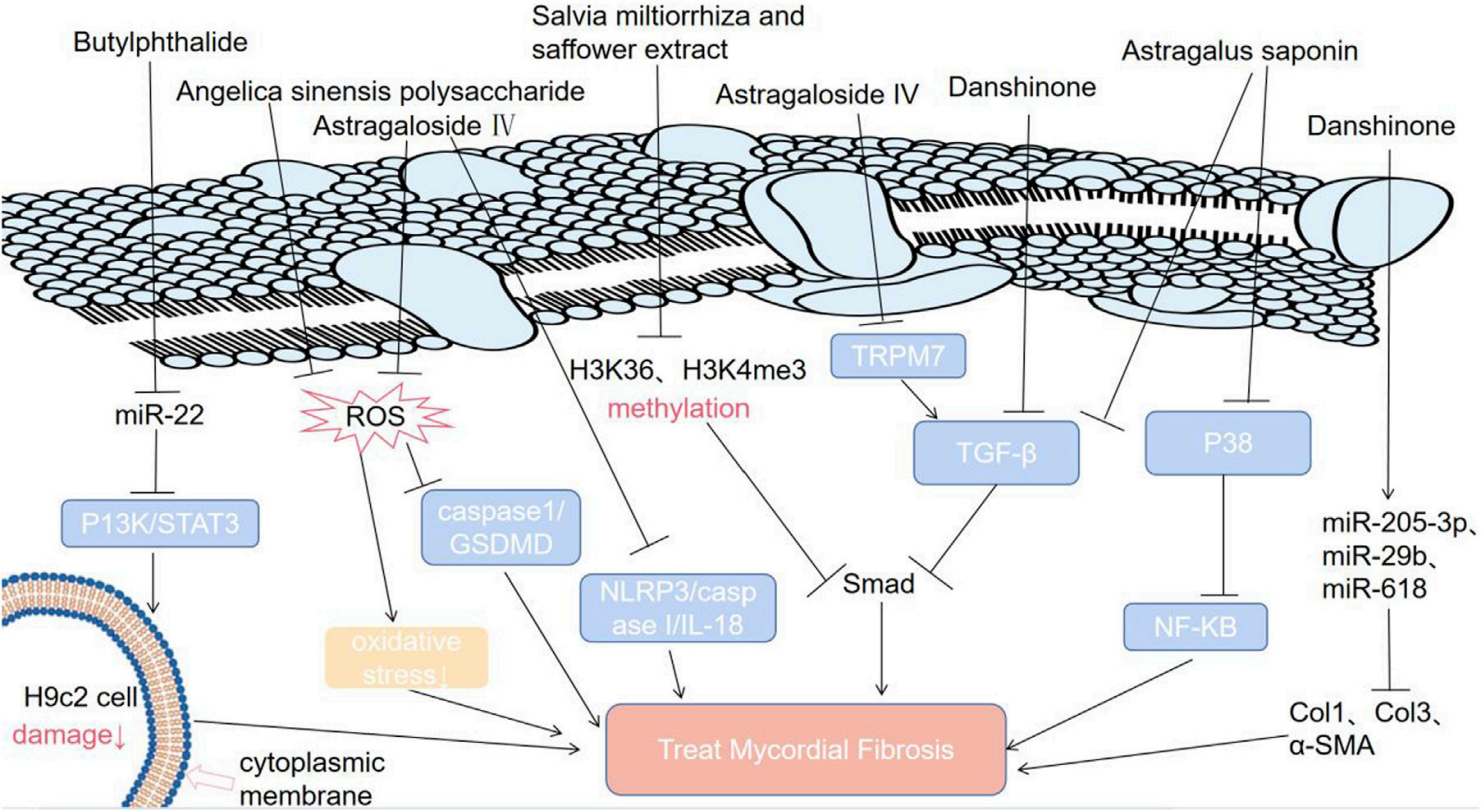
Molecular mechanisms by which TCM can treat MF.

A mixture of active ingredients from SCE can exert a synergistic effect (Orgah et al., 2020). This combined medication is better than individual medicines in terms of the therapeutic effects on cardiovascular diseases (Orgah et al., 2020). It was reported that the effect of SCE on attenuating fibrosis was closely linked with the downregulation of TGF-β/Smad3 signaling and that SCE can inhibit the increase in the levels of H3K4me3 and H3k36me3 in the Smad3 promoter region induced by TGF-β in CFs (Figure 3) (Yang et al., 2019). In addition, when the levels of water-soluble effective components (salvianolic acid B and hydroxysafflower yellow A) of SCE are increased, they can protect the damaged heart by inhibiting tissue oxidation, inflammatory cell infiltration and platelet aggregation (Orgah et al., 2020).

The results of experimental research and clinical practice have proven that SCE can exert a good therapeutic effect on MF, but potential targets and molecular mechanisms of its inhibitory effects on MF need to be further studied.

### 3.2 Astragalus

Astragalus is a promising antifibrotic drug (Ren et al., 2023). Its main antifibrotic components are calycosin, astragaloside IV, astragalus polysaccharides and formononetin (Tan et al., 2020) (Table 1). It has been confirmed that *astragalus membranaceus* and its effective components can inhibit MF (Pan and Zhu, 2018). Astragalus saponins can inhibit the TGF-β/Smad pathway and P38 MAPK/NF-KB pathway, alleviating MF (Figure 3) (Zhu et al., 2022). Astragaloside IV has four mechanisms (Pan et al., 2021; Li et al., 2023; Zhang et al., 2022; Wan et al., 2018; Wei et al., 2020; Li et al., 2021; Lu et al., 2017). First, astragaloside IV inhibits the TGF-β-smad signaling pathway to inhibit MF (Pan et al., 2021; Li et al., 2023). Second, astragaloside IV alleviates MF by suppressing the ROS/caspase1/gasdermin D (GSDMD) signaling pathway in mice (Figure 3) (Zhang et al., 2022). Third, astragaloside IV exerts antifibrotic effects by inhibiting the NOD-like receptor family pyrin domain-containing 3 (NLRP3)/caspase I/IL-18 pathway in mice with isoproterenol-induced cardiac fibrosis (Figure 3) (Wan et al., 2018). Fourth, astragaloside IV significantly downregulates the transient receptor potential melastatin 7 (TRPM7) channel to inhibit hypoxia-induced cardiac fibrosis. Astragaloside IV can alleviate cardiac fibrosis by targeting the mir-135a-TRPM7-TGF-β/Smad pathway (Figure 3) (Wei et al., 2020; Li et al., 2021; Lu et al., 2017).

It has been reported that *astragalus membranaceus* has therapeutic effects on MF, but its material basis remains unclear (Ren et al., 2023). Moreover, there are many research limitations. Technical limitations suggest that the exact components of *astragalus mongholicus* Bunge or the compound medicine cannot be fully determined. In addition, the exact therapeutic effect of *astragalus mongholicus* Bunge on MF cannot be accurately determined by a multicomponent study, and the results of randomized controlled trials demonstrating its efficacy are not sufficient (Ren et al., 2023).

### 3.3 Angelica

*Angelica* contains a variety of components that have a wide range of biological activities, such as immune regulation, liver protection, and antiatherosclerotic, antitumor, anti-inflammatory and analgesic activities (Table 1) (Chang et al., 2021).

It has been reported that butylphthalide, the active ingredient of *angelica*, can activate the P13K/STAT3 pathway by downregulating miR-22, reducing H9c2 cell damage and inhibiting MF during myocardial infarction under hypoxia *in vitro* (Figure 3) (Lin et al., 2019). *Angelica sinensis polysaccharide* (ASP), a major bioactive component extracted from the roots of angelica, has antioxidative activity and can treat multiple diseases resulting from oxidative stress (Song et al., 2021). ASP can decrease ROS levels in a dose-dependent manner (Song et al., 2021; Pan and Zhu, 2018). It has been shown that ASP can alleviate cardiac fibrosis by inhibiting oxidative stress (Song et al., 2021; Pan and Zhu, 2018). The underlying and detailed mechanisms by which ASP prevents MF are worthy of further study.

### 3.4 Other TCM

Other TCMs, such as curcumin, earthworms and ginsenoside, have therapeutic effects on MF (Yu et al., 2019; Lai et al., 2015; Wang et al., 2021). Curcumin decreases cardiac fibrogenesis by activating SIRT1, increasing Nrf2, and increasing NADPH oxidase subunits (Sadoughi et al., 2021; Ren et al., 2022). Earthworms can inhibit MF by inhibiting the levels of MF-related proteins in H9c2 cells (Huang et al., 2019). Ginsenoside Re, the active ingredient of ginsenoside, can alleviate MF by inhibiting the miR-489/MyD88/NF-ΚB signaling pathway (Table 2) (Sun et al., 2023).

Traditional Chinese medicine inhibits MF by downregulating some related signaling pathways (P13K/STAT3, TGF-β/Smad, NF-KB), related molecules (miR-22, H3K36, H3K4me3, miR-618), and levels of reactive oxygen species. ROS: reactive oxygen species; NF-kB: K gene binding nuclear factor; TRPM7: transient receptor potential melastatin 7; P38: an important stress activating member of the MAPK family; STAT3: signal transduction and activator of transcription; P13K: phosphoinositide 3 kinase; NLRP3: nucleotide binding oligomerization domain-like receptor protein 3; IL-18: interleukin-18; caspase 1: cysteinyl aspartate specific proteinase 1; Col1: collagen type 1; Col3: collagen type 3; H3K4me3: trimethylation of lysine in the third subunit of histone 4; H3K36me3: histone 3 lysine 36 trimethylation.

Other modern medicine and traditional Chinese medicine inhibit MF through relevant molecular mechanisms (ROS/NF-KB-Snail pathway, ERK1/2, uPA, SP1, CTGF proteins). SIRT1: Silencing regulatory protein 1; Nrf2: encoded by the NFE2L2 gene, regulating approximately 250 genes involving in cellular homeostasis; H9C2 cells: Embryonic rat cardiomyocytes; uPA: a multifunctional serine protease with a relative molecular weight of 55,000 kDa, which can be synthesized by fibroblasts, monocyte, neutrophils, epithelial cells, and tumor cells; SP1: sequence-specific DNA binding proteins that regulates the transcription of cellular and viral genes rich in GC sequences in certain promoters. It is an important and essential transcription factor. CTGF protein: connective tissue growth factor protein.

## 4 Conclusion and perspective

To date, increasing attention has been focused on MF. MF is closely linked with the occurrence and development of various heart diseases, including atherosclerosis, coronary disease and myocardial infarction. Modern medicine and TCM, two different medical systems, are important for treating MF and have their own advantages and disadvantages. Compared with TCM, modern medicine has experienced rigorous scientific testing and regulation, but it has potential side effects and high treatment costs. In addition, TCM is effective in treating MF, but because of its unclear composition and targeting site, it is difficult to elucidate the detailed mechanisms (Liu et al., 2022). Fundamental scientific studies, rigorous clinical trials, standard production regulation and quality control can promote the development and credibility of TCM worldwide. These processes can contribute to a better understanding of TCM and develop its clinical value in the treatment of MF in the future (Yang et al., 2022).

Collocation of TCM and modern medicine in treating MF has attracted more attention. The combination of Guanxinning injection (GXNI) and modern medical techniques can better treat MF (Fan et al., 2023). It is confirmed that GXNI can substantially alleviate MF via H&E and masson staining methods (Fan et al., 2023). In addtion, the relationship between transforming growth factor-beta receptor 1 (TGFBR1) and calycosin has been revealed through using modern medical techniques including molecular docking, molecular dynamics (MD) simulation and surface plasmon resonance imaging (SPRi) (Chen et al., 2022). It is revealed that calycosin can attenuate MF by downregualting the TGFBR1 signaling pathway (Chen et al., 2022). Modern medical technology can promote the application of TCM in the prevention and treatment of MF. The rapid development of modern medicine is both challenge and opportunity for the integration of TCM and modern medicine. Modern medical technology and achievements are beneficial for complementary advantages of TCM and modern medicine (Xu and Chen, 2007).

We reviewed and summarized the research progress of modern medicine and TCM to treat MF, which can provide more references and be beneficial for further study of the development of drugs to treat MF.
